# Clinical predictors of postoperative complications in the context of enhanced recovery (ERAS) in patients with esophageal and gastric cancer

**DOI:** 10.1007/s13304-023-01739-6

**Published:** 2024-02-15

**Authors:** Christian Geroin, Jacopo Weindelmayer, Serena Camozzi, Barbara Leone, Cecilia Turolo, Selma Hetoja, Maria Bencivenga, Michele Sacco, Carlo Alberto De Pasqual, Eugenia Mattioni, Giovanni de Manzoni, Simone Giacopuzzi

**Affiliations:** General and Upper G.I. Surgery Division, University of Verona, Borgo Trento, Verona, Italy

**Keywords:** Gastric and esophageal cancer, Enhanced recovery after surgery, Postoperative complications, Surgery, Prehabilitation

## Abstract

The overall frequency of postoperative complications in patients with esophageal and gastric cancer diverges between studies. We evaluated the frequency and assessed the relationship between complications and demographic and clinical features. For this observational study, data were extracted from the ERAS Registry managed by the University of Verona, Italy. Patients were evaluated and compared for postoperative complications according to the consensus-based classification and the Clavien–Dindo scale. The study population was 877 patients: 346 (39.5%) with esophageal and 531 (60.5%) with gastric cancer; 492 (56.2%) reported one or more postoperative complications, 213 (61.6%) of those with esophageal and 279 (52.5%) of those with gastric cancer. When stratified by consensus-based classification, patients with esophageal cancer reported general postoperative complications more frequently (p < 0.001) than those with gastric cancer, but there was no difference in postoperative surgical complications between the two groups. Multiple logistic regression models revealed an association between postoperative complications and the Charlson Comorbidity Index (adjusted odds ratio [OR] 1.22; 95% confidence interval [CI] 1.08–1.36), operation time (adjusted OR, 1.08; 95% CI 1.00–1.15), and days to solid diet intake (adjusted OR, 1.39; 95% CI 1.20–1.59). Complications in patients with esophageal and gastric cancer are frequent, even in those treated according to ERAS principles, and are often associated with comorbidities, longer operative time, and longer time to solid diet intake.

## Introduction

Postoperative complications in patients with esophagogastric cancer are associated with increased mortality, longer hospital length of stay (LOS), and higher healthcare costs [1–5]. The overall 30 days frequency in esophagogastric cancer varies, with morbidity rates from 24 to 59% in patients with esophageal cancer, with predominant pulmonary and cardiac complications [5–8], and from 10 to 40% in patients with gastric cancer [9, 10]. Previous studies investigating postoperative complications reported a wide range of incidence rates due to the use of inconsistent definitions and reporting systems (major, minor or overall complications), as well as various classifications (e.g., Clavien-Dindo, complications list) [2, 3, 6, 7, 11]. Recently, two international expert consensus classifications for complications have helped to improve consistency in their reporting in esophagectomy [5] and gastrectomy (intraoperative, postoperative general, postoperative surgical) [2], thereby facilitating comparison of outcomes.

Several studies have focused on reducing complications by improving surgical techniques [12]. Currently, perioperative care is being standardized through the implementation of the Enhanced Recovery After Surgery (ERAS) protocol [13, 14]. The application of ERAS protocols in esophagogastric cancer surgery [13, 14] has been associated with better outcomes and lower hospital costs as compared with conventional care [15–17]. Systematic reviews and meta-analyses in esophagectomy have reported fewer pulmonary complications and shorter hospital length of stay (LOS), without increasing readmissions [18–20]. Similarly, the implementation of ERAS in gastrectomy has demonstrated a reduction in intraabdominal drain and nasogastric tube usage, facilitated a faster return to solid diet, and resulted in shorter LOS, without increasing complications [12]. Despite improvements in postoperative care and surgical techniques, surgery is still associated with high morbidity rates ranging from 26 to 64% in esophageal cancer [18, 19] and from 5 to 33.9% in gastric cancer [15, 21, 22]. While this raises questions about the benefit of ERAS for reducing postoperative morbidity [23], the studies may not have adequately captured the true incidence rate of complications for several reasons. First, they may have underestimated the rate by considering only “major” complications. Second, they did not classify complications as proposed by the international consensus [18, 19], which limits comparison of incidence rates among study canters. Moreover, these cohorts did not consistently report adherence to the ERAS program, except for one study [15]. Because adherence to the entire ERAS pathway is crucial for obtaining the greatest benefits, any lack of adherence could increase the complications rate. Finally, many of these cohorts were retrospective studies [18, 21, 22], which may have introduced potential referral bias. They also failed to compare between the clinical and demographic characteristics of patients with and without complications and those with esophageal and gastric cancer.

To overcome these possible limitations, we conducted a prospective study using the ERAS Registry of our Institution and applied an international classification of complications [2]. We reported the level of adherence to ERAS [15] and provided a detailed description of several clinical features. The aim was to assess and compare the complications rates in a large patient cohort with esophageal and gastric cancer and to examine the relationship with demographic and clinical variables. Our findings may be useful for identifying at-risk patients and complications-associated comorbidities that could benefit from earlier therapeutic strategies (prehabilitation).

## Methods

For this observational study, data were extracted from the ERAS Registry managed by the General and Upper G.I. Surgery Division, University of Verona, Verona, Italy. The ERAS Registry prospectively collects data on symptoms, natural history, risk factors, and comorbidity in patients with esophageal or gastric cancer [15].

### Subjects

The study population was patients with a diagnosis of esophageal, esophagogastric junction, and gastric cancer who underwent surgery with radical intent in the context of an ERAS program [15] at our Institution between November 2013 and December 2022. Exclusion criteria were resection performed without curative intent, multiorgan resection, and urgent procedures.

We collected the following clinical, oncological, surgical data, and postoperative complications [4]: age, sex, body-mass index (BMI, weight in kg divided by height in meters squared), smoking history, serum albumin level, preoperative comorbidities, the Charlson Comorbidities Index (CCI) calculated without including the esophageal and gastric cancer score, history of major surgery, American Society of Anesthesiologists (ASA) score, tumor histology, location, and TNM pathological stage. We also recorded neoadjuvant therapy, type of surgery, intra-operatory infusion, operative time, days to ward transfer, days to sitting/standing upright, days to independent gait without external aid, nutrition intake, and days to hospital discharge. Occurrence of one or more postoperative complications was recorded up to 90 days after the operation [15] according to a consensus-based classification (postoperative general, postoperative surgical complications) [2] and the Clavien–Dindo scale (CD). Compliance with the protocol was evaluated according to the 15 ERAS items [15, 17].

### Statistical analysis

Data are expressed as mean ± standard deviation (SD) and range for continuous variables, counts, and percentages for categorical variables. We compared the groups using the Mann–Whitney U test for continuous variables and the chi-squared test or Fisher’s exact test (if ≤ 5 expected frequencies) for categorical variables. Logistic regression models were created to estimate unadjusted and adjusted odds ratio (OR; 95% confidence interval [CI]) for one or more complications (dependent variable) in relation to sociodemographic and clinical characteristics (independent variables). Independent variables were chosen according to exploratory analysis results and clinical relevance. All tests were statistically significant at p < 0.05. Statistical analysis was performed using SPSS statistical software version 20 (IBM-SPSS, Armonk, NY, USA).

## Results

### Demographic, surgical, and post-operative data of total sample

**Fig. 1:**
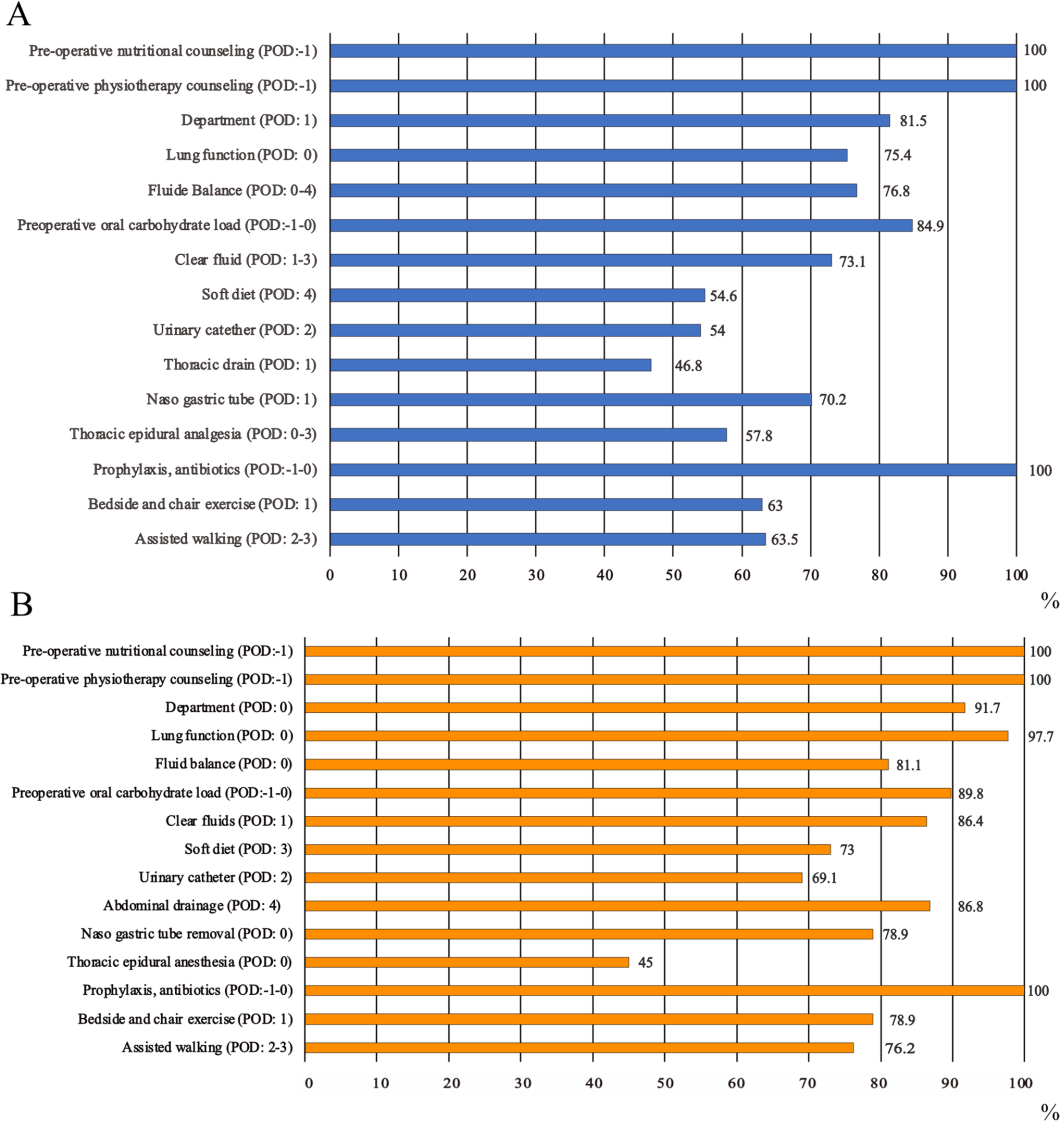
Compliance with ERAS items (n = 15) for esophageal (panel **A**) and gastric (panel **B**) cancer; *POD* denotes postoperative days

The study population was 877 patients, 346 (39.5%) treated for esophageal and 531 (60.5%) for gastric cancer; 615 (70.1%) were male and 262 (29.9%) were female, with a mean age of 68.7 ± 12.6 years. A total of 648 (73.8%) patients presented one or more preoperative comorbidities: cardiovascular (n = 486, 55.4%), respiratory (n = 133, 15.2%), metabolic (n = 226, 25.8%), and kidney (n = 59, 6.7%) disease, with an average of CCI of 1.2 ± 1.5. Previous major surgery was reported in 154 (17.6%) patients. The ASA score was II in 537 (61.5%). Adenocarcinoma was the most frequent type of tumor (overall, n = 750, 85.8%) with pathological stage T grade 3 in 234 (27.4%), stage N grade 0 in 406 (47.8%), and stage M grade 0 in 782 (91.7%). The majority of patients (n = 530, 60.6%) underwent neoadjuvant therapy. Different types of surgery were employed: Ivor-Lewis was the most frequent technique (n = 251, 64.5%) in patients with esophageal cancer, while total gastrectomy was the most frequent (n = 290, 59.9%) in patients with gastric cancer. The total average amount of intraoperative infusion was 3129.4 ± 1578.3 mL, the operative time was 6.6 ± 2.6 h, the time to ward transfer was 0.5 ± 1.4 days, the time to position the patient in sitting/standing position by the physiotherapist was 1.4 ± 1.7 days, and the time to independent unaided gait was 3.1 ± 3.2 days. The carbohydrate load was delivered in 771 (88.2%) patients. The time to return to solid diet was 4.8 ± 4.7 days, and hospital discharge occurred at 10.2 ± 8.8 days on average (Table 1). Adherence to ERAS after its implementation was appropriate for many items, with a compliance rate of 70% or higher for 9 and 14 out of 15 items for esophageal and gastric cancer, respectively (Fig. 1, panel A, B).

**Table 1:** Demographic and clinical features of patients with esophageal or gastric cancer with or without postoperative complications

Variable	Total sample	Without complications	With complications	Without vs. with complications (p value)
Patients, no, %	877	385 (43.8)	492 (56.2)	
Sex, no. (%)				
Male	615 (70.1)	259 (67.3)	356 (72.4)	0.103^b^
Female	262 (29.9)	126 (32.7)	136 (27.6)	
Age, year, mean (SD)	68.7 (12.6)	66.9 (12.7)	70.1 (12.4)	** < 0.001**^d^
Body-mass index (kg/m^2^), mean (SD)	25.1 (4.3)	25 (4.2)	25.2 (4.4)	0.864^d^
Smoking history, no. (%)	141 (16.1)	53 (13.8)	88 (17.9)	0.099^b^
Serum albumin level, g/L, mean (SD)	38.02 (6.3)	38.2 (5.8)	37.8 (6.6)	0.329^d^
Preoperative comorbidities, no. (%)				
Cardiovascular	486 (55.4)	188 (48.8)	298 (60.6)	**0.001**^**b**^
Respiratory	133 (15.2)	43 (11.2)	90 (18.3)	**0.004**^**b**^
Metabolic diseases	226 (25.8)	84 (21.8)	142 (28.9)	**0.018**^**b**^
Kidney	59 (6.7)	21 (5.5)	38 (7.7)	0.183^b^
Charlson comorbidity index, mean (SD)	1.2 (1.5)	0.8 (1.2)	1.4 (1.6)	** < 0.001**^**d**^
Previous major surgery, no. (%)	154 (17.6)	57 (14.8)	97 (19.7)	0.058^b^
American Society of Anesthesiologists score (%)				
I	32 (3.7)	21 (5.5)	11 (2.2)	**0.012**^**b**^
II	537 (61.5)	264 (68.8)	273 (55.8)	** < 0.001**^**b**^
III	279 (32)	92 (24)	187 (38.2)	** < 0.001**^**b**^
IV	25 (2.9)	7 (1.8)	18 (3.7)	0.102^b^
Cancer characteristics				
Esophageal	346 (100)	133 (38.4)	213 (61.6)	**0.009**^**b**^
Gastric	531 (100)	252 (47.5)	279 (52.5)	
Tumor histology, no. (%)				
Adenocarcinoma	750 (85.8)	350 (91.4)	400 (81.5)	** < 0.001**^**b**^
Squamous	99 (11.3)	23 (6)	76 (15.5)	** < 0.001**^**b**^
Mixed	4 (0.5)	0	4 (0.8)	0.136^c^
NET/Others	21 (2.4)	10 (2.6)	11 (2.2)	0.723^b^
Tumor location, no. (%)				
Esophagus	251 (28.9)	96 (25.2)	155 (31.8)	**0.034**^**b**^
Esophagogastric junction	235 (27)	92 (24.1)	143 (29.3)	0.090^b^
Stomach	383 (44.1)	193 (50.7)	190 (38.9)	**0.001**^**b**^
Pathological stage (T)				
0	121 (14.2)	56 (15)	65 (13.4)	0.541^b^
I	156 (18.3)	71 (19)	85 (17.7)	0.619^b^
II	112 (13.2)	51 (13.7)	61 (12.7)	0.679^b^
III	234 (27.4)	97 (26)	137 (28.3)	0.449^b^
IV	230 (27)	98 (26.3)	132 (27.9)	0.689^b^
Pathological stage (N)				
0	406 (47.6)	174 (46.6)	232 (48.3)	0.625^b^
I	160 (18.8)	71 (19)	89 (18.5)	0.855^b^
II	109 (12.8)	45 (12.4)	64 (13.3)	0.582^b^
III	173 (20.8)	82 (22)	91 (19.9)	0.276^b^
Pathological stage (M)				
0	782 (91.7)	348 (93.3)	434 (90.4)	0.131^b^
I	71 (8.3)	25 (6.7)	46 (9.6)	0.131^b^
Neoadjuvant therapy, no. (%)	530 (60.6)	228 (59.4)	302 (61.6)	0.498^b^
Type of surgery, no. (%)				
*Esophagus*				
Hybrid or mini-invasive intervention	166 (48.1)	49 (37.1)	117 (54.9)	**0.001**^**b**^
Open	179 (51.9)	83 (62.9)	96 (45.1)	
*Stomach*^a^				
Mini-invasive intervention	62 (12.8)	35 (14.9)	27 (10.8)	0.183^b^
Open	422 (87.2)	200 (85.1)	222 (89.2)	
Type of surgery, no. (%)				
*Esophagus*				
Ivor-Lewis	251 (64.5)	109 (73.7)	142 (58.9)	**0.005**^**b**^
McKeown	84 (21.6)	20 (13.5)	64 (26.6)	
Esophagogastrectomy^b^	54 (13.9)	19 (12.8)	35 (14.5)	
*Stomach*				
Total gastrectomy	290 (59.9)	143 (60.9)	147 (59)	0.684^c^
Subtotal gastrectomy	194 (40.1)	92 (39.1)	102 (41)	
Intra-operatory infusion, mL	3129.4 (1578.3)	2798.20(1049.9)	3381.93 (1845.8)	** < 0.001**^**d**^
Operation time, hours, mean (SD)	6.6 (2.6)	6.1 (2.3)	7.02 (2.7)	** < 0.001**^**M**^
Ward transfer, days, mean (SD)	0.5 (1.4)	0.3 (0.6)	0.7 (1.8)	** < 0.001**^**d**^
Physiotherapy				
Verticalization, days, mean (SD)	1.4 (1.7)	1.2 (0.6)	1.6 (2.3)	** < 0.001**^**d**^
Independent gait, days, mean (SD)	3.1 (3.2)	2.4 (1.2)	3.6 (4.1)	** < 0.001**^**d**^
Nutrition				
Carbohydrate load, no. (%)	771 (88.2)	339 (88.1)	432 (88.3)	0.894^b^
Liquid diet, days mean (SD)	2.05 (3.4)	1.4 (1.2)	2.5 (4.4)	** < 0.001**^**d**^
Solid diet, days mean (SD)	4.8 (4.7)	3.6 (1.2)	5.8 (6.1)	** < 0.001**^**d**^
Hospital discharge, days mean (SD)	10.2 (8.8)^**d**^	6.7 (1.5)	12.9 (11.1)	** < 0.001**^**d**^

*Abbreviations: SD* standard deviation, *NET* neuroendocrine tumor; Other interventions: Ivor-Lewis, McKeown, esophagogastrectomy for esophageal surgery; other interventions total gastrectomy and subtotal gastrectomy for stomach cancer

^a^Stomach does not include n = 46 patients who underwent esophagectomy; Mini-invasive for both esophagus and stomach denotes laparoscopic surgery

^b^Chi-square test

^c^Fisher’s exact test

^d^Mann–Whitney U Test; in bold: statistically significant values with p < 0.05. Missing: serum albumin level n = 20; Charlson Comorbidity Index n = 23; American Society of Anesthesiologists score n = 4; tumor histology n = 3; tumor location n = 8; clinical stage (t) n = 24; clinical stage (n) n = 29; clinical stage (m) n = 24; neoadjuvant therapy n = 3; Type of surgery, esophagus, hybrid or mini-invasive vs open n = 1; stomach, hybrid or mini-invasive vs open n = 47

^e^Esophagectomy: includes n = 46 patients with gastric cancer, plus 3 missing values; Ivor-Lewis n = 1; McKeown n = 1; esophagogastrectomy n = 1; minimally invasive esophagectomy n = 1; total gastrectomy n = 47; subtotal gastrectomy n = 47; minimally invasive gastrectomy n = 47; intra-operatory infusion n = 105; operative time n = 4; ward transfer n = 4; verticalization n = 14; independent gait n = 23; carbohydrate load n = 3; liquid diet = 11; solid diet n = 19; hospital discharge n = 23

### Demographic and clinical features of patients with and without complications

**Fig. 2:**
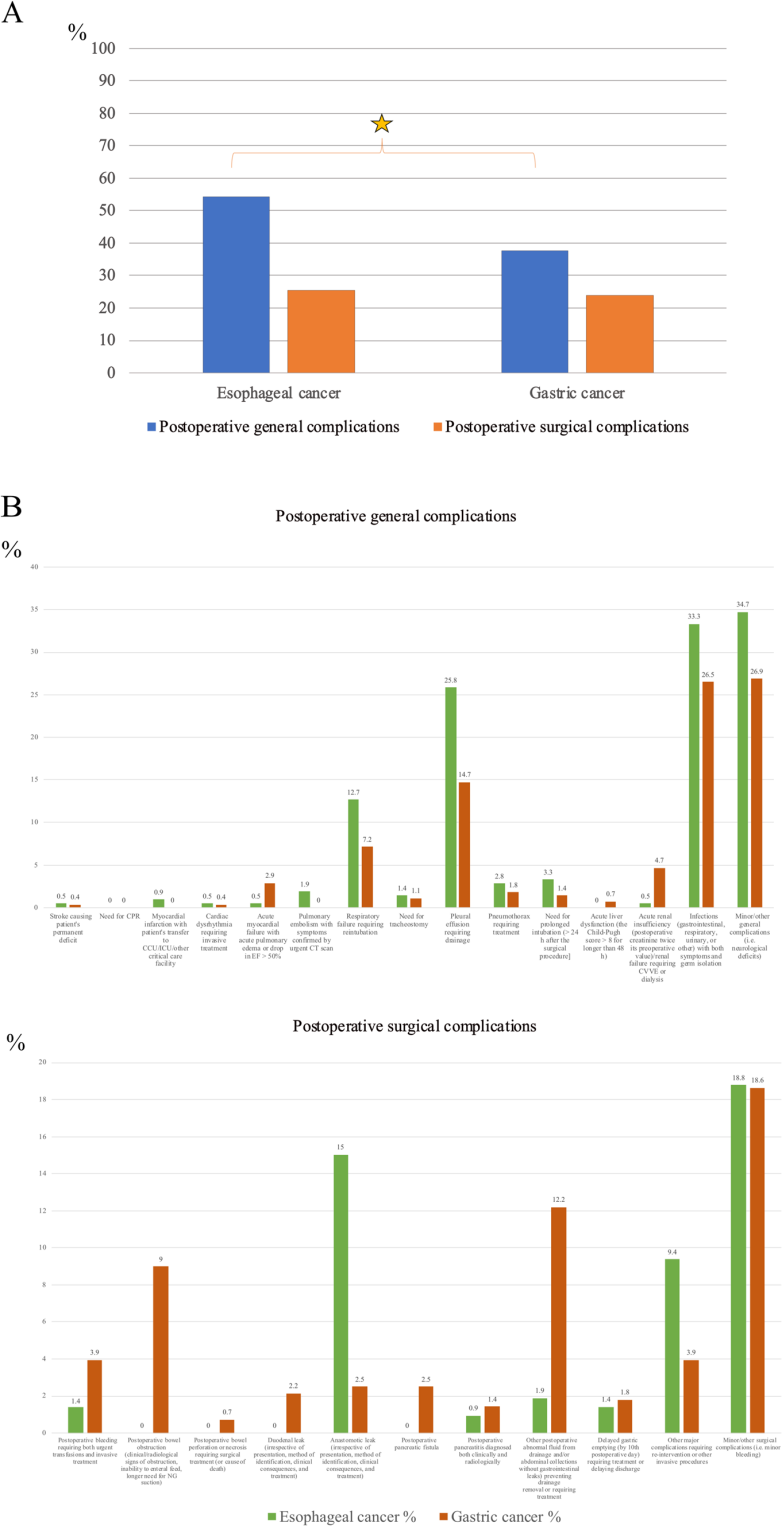
Panel **A** Comparison of complications in patients treated for esophageal or gastric cancer according to a consensus-based classification; panel **B** percentage of postoperative general and surgical complications. The category “minor/other” includes complications that cannot be classified within the consensus-based classification. Abbreviations: *CPR* cardiopulmonary resuscitation, *CCU* coronary care unit, *ICU* intensive care unit, *EF* ejection fraction, *CVVH* continuous veno-venous hemofiltration, *NG* nasogastric

We found that 492 (56.2%) patients reported one or more postoperative complications: 213 (61.6%) of those treated for esophageal and 279 (52.5%) of those treated for gastric cancer. When stratified by the consensus-based classification, the patients with esophageal cancer reported postoperative general complications more frequently (54%, p < 0.001) than those with gastric cancer (37%). No difference in postoperative surgical complications was noted between those with esophageal (25.5%) and gastric cancer (23.8%) (Fig. 2, panel A). Only one isolated general complication was recorded in 133 (71.1%) patients with esophageal cancer, while a combined form (more than one general complication) was recorded in 54 (28.9%). Only one isolated surgical complication was recorded in 73 (83%) patients, while a combined form (more than one surgical complication was recorded in 15 (17%). Only one isolated general complication was record in 158 (79.7%) patients with gastric cancer, while a combined form (more than one general complication was record in 40 (20.3%). Only one isolated surgical complication was recorded in 92 (73.6%), while a combined form (more than one surgical complication) was recorded in 33 (26.4%). A complete list of complications is presented in Fig. 2, panel B.

Patients with esophageal cancer reported grade IIIa and IVa complications more frequently but fewer grade I complications than those with gastric cancer (CD score, Table 2). In addition, patients with postoperative complications were older, had preoperative comorbidities more often (e.g., cardiovascular, respiratory, metabolic), a higher CCI score, ASA score grade III, greater amount of intraoperative liquid infusion, and a more frequent squamous tumor histology located predominately in the esophagus than patients without postoperative complications. Patients with postoperative complications and esophageal tumor underwent mini-invasive intervention and Ivor-Lewis surgery more frequently. They were also noted to have longer operative time and more days to ward transfer, stand/sit upright and walk unaided, return to liquid/solid diet intake, and hospital discharge than patients without complications (Table 1).

**Table 2:** Clavien-Dindo classification of surgical complications in patients with esophageal or gastric cancer

Variable	Total sample	Esophageal cancer	Gastric cancer	p value
Patients, no. (%)	478	204	274	
I	71 (8.1)	19 (5.5)	52 (9.8)	**0.026**^a^
II	212 (24.2)	77 (22.3)	135 (25.4)	0.340^a^
III	2 (0.2)	0	2 (0.4)	0.523^b^
IIIa	120 (13.7)	65 (18.8)	55 (10.4)	** < 0.001**^a^
IIIb	27 (3.1)	13 (3.8)	14 (2.6)	0.327^a^
IV	1 (0.1)	0	1 (0.2)	1000^b^
IVa	36 (4.1)	26 (7.5)	10 (1.9)	** < 0.001**^**a**^
IVb	2 (0.2)	1 (0.3)	1 (0.2)	1000^b^
V	7 (0.8)	3 (0.9)	4 (0.8)	1000^b^

In bold: statistically significant values

^a^Chi-square test

^b^Fisher’s exact test; Missing: n = 14 (n = 9 esophageal cancer, n = 5 stomach cancer)

The univariate logistic regression model yielded an association between postoperative complications and many of clinical and demographic features (Table 1). After adjusting for all variables in the model, multivariate logistic regression analysis confirmed associations with the variables: CCI (adjusted OR, 1.22; 95% CI 1.08–1.36), operative time (adjusted OR, 1.08; 95% CI 1.00–1.15), and number of days to return to solid diet intake (adjusted OR, 1.39; 95% CI 1.20–1.59) (Table 3).

**Table 3:** Clinical and demographic variables associated with postoperative complications in patients with esophageal or gastric cancer

	Adjusted		
Independent variable	OR	95% CI	p value
Age, year	1.01	0.99–1.02	0.084
Charlson comorbidity index, score	1.22	1.08–1.36	**0.001**
ASA III, score	1.39	0.97–1.98	0.066
Operative time, h	1.08	1.00–1.15	**0.034**
Ward transfer, days	0.89	0.68–1.17	0.441
Verticalization, days	1.13	0.81–1.56	0.468
Independent gait, days	1.05	0.89–1.23	0.561
Solid diet, days	1.39	1.20–1.59	** < 0.001**

Significant associations at p < 0.05; Bold indicates statistically significant values. *OR* odds ratio, *CI* confidence interval, *ASA* American Society of Anesthesiologists score; in bold: statistically significant values. Missing: Charlson Comorbidity Index (n = 23); American Society of Anesthesiologists score (n = 4); operative time (n = 4); ward transfer (n = 4); verticalization(n = 14); independent gait (n = 23; solid diet (n = 19)

## Discussion

In this observational study of the ERAS Registry, we found that one or more postoperative complications were recorded for 56.2% of patients with esophageal and gastric cancer. When stratified by a consensus based-classification [2], postoperative general complications were more frequent and severe in patients with esophageal cancer. We noted several associations between postsurgical complications and clinical and demographic features; however, few remained after we adjust for all variables in the model, such as higher CCI score, longer operative time, and more days to return to solid diet.

Despite improvement in postoperative care and surgical techniques achieved in ERAS program, surgery is still associated with high morbidity rates in both esophageal [18, 19] and gastric cancer [15, 21, 22]. It may be, possible, however, that the studies underestimated complications rates because they focused on “major” complications. In our cohort, the overall incidence of postoperative complications was slightly higher for the patients treated for gastric cancer, as we included all types of complications, including those with CD < 3. These data extend previous findings and suggest that complications are frequent in patients with esophageal and gastric cancer also in those treated according to ERAS principles. For example, esophagectomy was associated with a higher morbidity rate than gastrectomy, despite major advances in surgery, anesthesia, and perioperative care, including minimally invasive procedures.

We noted that patients with complications were more likely to have multiple comorbidities. Indeed, cardiovascular, respiratory, and metabolic comorbidities (e.g., diabetes mellitus) are frequent in patients with esophageal and gastric cancer [6, 24]. Previous studies suggested that patients with comorbidities and those using polypharmacy (≥ 5 medications) are more susceptible to developing complications [24]. Heart failure, hypertension, and renal insufficiency are reported to be independent clinical predictors associated with major complications and anastomotic leakage [24]. Vascular disease, particularly arterial calcification, is an independent predictor for severe complications, especially anastomotic leakage [24]. In patients undergoing laparoscopic total gastrectomy, the impact of comorbidities is considerable. For instance, pulmonary disease is associated with a higher risk of postoperative complications [25]. These data may also provide an explanation for the observed association between complications and longer operative time in our cohort.

The impact of operative time is likely to be experienced mostly by patients with poor physical and nutritional status [6]. Longer operative time is also a component of complex surgical intervention. The benefits of minimally invasive surgery notwithstanding (e.g., reduced pain), many studies have found that the procedure involves a steep learning curve, which increases operative time and may also lead to differences in outcomes between trainees and experienced surgeons.

We noted that patients with complications need more time before returning to a solid diet. One explanation for the delay in resuming solid food intake is that surgeons and nurses may be reluctant to initiate early oral feeding since patients with esophageal and gastric cancer may be at high risk of anastomosis leakage [26]. However, ERAS consensus guidelines recommend offering patients drink and food at will from day 1 after total gastrectomy [14]. These findings suggest the need to implement prehabilitation of nutrition support and physiotherapy, which could benefit patient well-being during perioperative periods [27]. Proper postoperative care for optimizing pulmonary function may be required for patients with pulmonary disease [25].

Adherence to the entire ERAS pathway is crucial for achieving the greatest benefits, and any lack of adherence may increase the complications rate. Except for one study [15], other previous studies did not consistently report adherence to the ERAS program In our study, all our patients were treated according to ERAS principles [13, 14], with a 70% adherence rate or higher for many items. Nonetheless, we observed no decrease in complications rates when compared with outcomes in a non-ERAS program. This observation is shared by previous studies that suggested that the ERAS program does not increase the complications rate as compared with conventional care but rather leads to better outcomes, reducing LOS [12] and hospital costs [15–17]. Despite the high adherence, there are plausible reasons for the lack of reduction of morbidity rate: heterogeneity/scant standardization in the number and the definition of ERAS components, resulting in low adherence by clinicians [23]; the unclear contribution of each item to prevent complications from arising; and finally, the difference in associated comorbidities, not all of which eligible for adherence to the ERAS program [28].

The present study has several limitations. First, our sample was made up of a mixed population. Esophageal cancer requires more complex invasive surgical procedures than gastric cancer, predisposing patients to more severe complications. Stratification based on type of cancer and severity (CD < 3B vs. CD ≥ 3B for esophageal and CD < 3A vs. CD ≥ 3A for gastric cancer) might have revealed further clinical predictors specific for each disease. Second, we stratified our sample by a consensus-based classification conceptualized for gastric [2] but not for esophageal cancer.

These limitations notwithstanding, our study findings indicate that postoperative complications are frequent in patients with esophageal and gastric cancer, and that they are often associated with comorbidities, longer operative time, and longer time to solid diet intake. Such patients may benefit from prehabilitation and from nutrition support and physiotherapy in particular.

## References

[1] Selby LV, Gennarelli RL, Schnorr GC, Solomon SB, Schattner MA, Elkin EB, Bach PB, Strong VE (2017) Association of hospital costs with complications following total gastrectomy for gastric adenocarcinoma. JAMA Surg 152(10):953–958 28658485 10.1001/jamasurg.2017.1718 PMC5710284

[2] Baiocchi GL, Giacopuzzi S, Marrelli D, Reim D, Piessen G, Matos da Costa P, Reynolds JV, Meyer HJ, Morgagni P, Gockel I, Lara Santos L, Jensen LS, Murphy T, Preston SR, Ter-Ovanesov M, Fumagalli Romario U, Degiuli M, Kielan W, Monig S, Kolodziejczyk P, Polkowski W, Hardwick R, Pera M, Johansson J, Schneider PM, de Steur WO, Gisbertz SS, Hartgrink H, van Sandick JW, Portolani N, Holscher AH, Botticini M, Roviello F, Mariette C, Allum W, De Manzoni G (2019) International consensus on a complications list after gastrectomy for cancer. Gastric Cancer 22(1):172–189 29846827 10.1007/s10120-018-0839-5

[3] Baiocchi GL, Giacopuzzi S, Vittimberga G, De Pascale S, Pastorelli E, Gelmini R, Vigano J, Graziosi L, Vagliasindi A, Rosa F, Steccanella F, Demartini P, Reddavid R, Berselli M, Elmore U, Romario UF, Degiuli M, Morgagni P, Marrelli D, D’Ugo D, Rosati R, De Manzoni G (2022) Clinical outcomes of patients with complicated post-operative course after gastrectomy for cancer: a GIRCG study using the GASTRODATA registry. Updates Surg. 10.1007/s13304-022-01318-1 35788552 10.1007/s13304-022-01318-1 PMC9852164

[4] Baiocchi GL, Giacopuzzi S, Reim D, Piessen G, Costa PMD, Reynolds JV, Meyer HJ, Morgagni P, Gockel I, Santos LL, Jensen LS, Murphy T, D’Ugo D, Rosati R, Fumagalli Romario U, Degiuli M, Kielan W, Monig S, Kolodziejczyk P, Polkowski W, Pera M, Schneider PM, Wijnhoven B, de Steur WO, Gisbertz SS, Hartgrink H, van Sandick JW, Botticini M, Holscher AH, Allum W, De Manzoni G (2020) Incidence and Grading of Complications After Gastrectomy for Cancer Using the GASTRODATA Registry: A European Retrospective Observational Study. Ann Surg 272(5):807–813 32925254 10.1097/SLA.0000000000004341

[5] Low DE, Kuppusamy MK, Alderson D, Cecconello I, Chang AC, Darling G, Davies A, D’Journo XB, Gisbertz SS, Griffin SM, Hardwick R, Hoelscher A, Hofstetter W, Jobe B, Kitagawa Y, Law S, Mariette C, Maynard N, Morse CR, Nafteux P, Pera M, Pramesh CS, Puig S, Reynolds JV, Schroeder W, Smithers M, Wijnhoven BPL (2019) Benchmarking complications associated with esophagectomy. Ann Surg 269(2):291–298 29206677 10.1097/SLA.0000000000002611

[6] Liu XL, Wang RC, Liu YY, Chen H, Qi C, Hu LW, Yi J, Wang W (2021) Risk prediction nomogram for major morbidity related to primary resection for esophageal squamous cancer. Medicine (Baltimore) 100(31):e26189 34397790 10.1097/MD.0000000000026189 PMC8341312

[7] Jezerskyte E, van Berge Henegouwen MI, van Laarhoven HWM, van Kleef JJ, Eshuis WJ, Heisterkamp J, Hartgrink HH, Rosman C, van Hillegersberg R, Hulshof M, Sprangers MAG, Gisbertz SS, Dutch Upper GICG (2021) Postoperative complications and long-term quality of life after multimodality treatment for esophageal cancer: an analysis of the prospective observational cohort study of esophageal-gastric cancer patients (POCOP). Ann Surg Oncol 28(12):7259–7276 34036429 10.1245/s10434-021-10144-5 PMC8519926

[8] Liang Z, Luo K, Wang Y, Zeng Q, Ling X, Wang S, Dragomir MP, Li Q, Yang H, Xi M, Chen B (2023) Clinical and dosimetric predictors for postoperative cardiopulmonary complications in esophageal squamous cell carcinoma patients receiving neoadjuvant chemoradiotherapy and surgery. Ann Surg Oncol 30(1):529–538 36127527 10.1245/s10434-022-12526-9

[9] Strong VE, Song KY, Park CH, Jacks LM, Gonen M, Shah M, Coit DG, Brennan MF (2010) Comparison of gastric cancer survival following R0 resection in the United States and Korea using an internationally validated nomogram. Ann Surg 251(4):640–646 20224369 10.1097/SLA.0b013e3181d3d29b

[10] Brenkman HJF, Gisbertz SS, Slaman AE, Goense L, Ruurda JP, van Berge Henegouwen MI, van Hillegersberg R (2017) Dutch upper gastrointestinal cancer audit, postoperative outcomes of minimally invasive gastrectomy versus open gastrectomy during the early introduction of minimally invasive gastrectomy in the netherlands: a population-based cohort study. Ann Surg 266(5):831–838 28742708 10.1097/SLA.0000000000002391

[11] Yu H, Xu L, Yin S, Jiang J, Hong C, He Y, Zhang C (2022) Risk factors and prognostic impact of postoperative complications in patients with advanced gastric cancer receiving neoadjuvant chemotherapy. Curr Oncol 29(9):6496–6507 36135080 10.3390/curroncol29090511 PMC9498105

[12] Salvans S, Grande L, Dal Cero M, Pera M (2023) State of the art of enhanced recovery after surgery (ERAS) protocols in esophagogastric cancer surgery: the Western experience. Updates Surg 75(2):373–382 35727482 10.1007/s13304-022-01311-8

[13] Low DE, Allum W, De Manzoni G, Ferri L, Immanuel A, Kuppusamy M, Law S, Lindblad M, Maynard N, Neal J, Pramesh CS, Scott M, Mark Smithers B, Addor V, Ljungqvist O (2019) Guidelines for Perioperative care in esophagectomy: enhanced recovery after surgery (ERAS((R))) society recommendations. World J Surg 43(2):299–330 30276441 10.1007/s00268-018-4786-4

[14] Mortensen K, Nilsson M, Slim K, Schafer M, Mariette C, Braga M, Carli F, Demartines N, Griffin SM, Lassen K (2014) Enhanced Recovery After Surgery, Consensus guidelines for enhanced recovery after gastrectomy: enhanced Recovery After Surgery (ERAS(R)) Society recommendations. Br J Surg 101(10):1209–29 25047143 10.1002/bjs.9582

[15] Weindelmayer J, Mengardo V, Gasparini A, Sacco M, Torroni L, Carlini M, Verlato G, de Manzoni G (2021) Enhanced recovery after surgery can improve patient outcomes and reduce hospital cost of gastrectomy for cancer in the west: a propensity-score-based analysis. Ann Surg Oncol 28(12):7087–7094 33988796 10.1245/s10434-021-10079-x PMC8519899

[16] Weindelmayer J, Verlato G, Alberti L, Poli R, Priolo S, Bovo C, de Manzoni G (2019) Enhanced recovery protocol in esophagectomy, is it really worth it? A cost analysis related to team experience and protocol compliance. Dis Esophagus. 10.1093/dote/doy114 30496453 10.1093/dote/doy114

[17] Giacopuzzi S, Weindelmayer J, Treppiedi E, Bencivenga M, Ceola M, Priolo S, Carlini M, de Manzoni G (2017) Enhanced recovery after surgery protocol in patients undergoing esophagectomy for cancer: a single center experience. Dis Esophagus 30(4):1–6 28375472 10.1093/dote/dow024

[18] Findlay JM, Gillies RS, Millo J, Sgromo B, Marshall RE, Maynard ND (2014) Enhanced recovery for esophagectomy: a systematic review and evidence-based guidelines. Ann Surg 259(3):413–431 24253135 10.1097/SLA.0000000000000349

[19] Markar SR, Karthikesalingam A, Low DE (2015) Enhanced recovery pathways lead to an improvement in postoperative outcomes following esophagectomy: systematic review and pooled analysis. Dis Esophagus 28(5):468–475 24697876 10.1111/dote.12214

[20] Puccetti F, Wijnhoven BPL, Kuppusamy M, Hubka M, Low DE (2022) Impact of standardized clinical pathways on esophagectomy: a systematic review and meta-analysis. Dis Esophagus. 10.1093/dote/doab027 34009322 10.1093/dote/doab027

[21] Desiderio J, Stewart CL, Sun V, Melstrom L, Warner S, Lee B, Schoellhammer HF, Trisal V, Paz B, Fong Y, Woo Y (2018) Enhanced recovery after surgery for gastric cancer patients improves clinical outcomes at a US Cancer center. J Gastric Cancer 18(3):230–241 30276000 10.5230/jgc.2018.18.e24 PMC6160527

[22] Blumenthaler AN, Robinson KA, Kruse BC, Munder K, Ikoma N, Mansfield PF, Gottumukkala V, Kapoor R, Badgwell BD (2021) Implementation of a perioperative-enhanced recovery protocol in patients undergoing open gastrectomy for gastric cancer. J Surg Oncol 124(5):780–790 34227691 10.1002/jso.26591 PMC8429131

[23] Tang Z, Lu M, Qu C, Zhang Y, Li L, Li S, Qi L, Cheng C, Tian H (2022) Enhanced recovery after surgery improves short-term outcomes in patients undergoing esophagectomy. Ann Thorac Surg 114(4):1197–1204 34624264 10.1016/j.athoracsur.2021.08.073

[24] van Kooten RT, Bahadoer RR, Peeters K, Hoeksema JHL, Steyerberg EW, Hartgrink HH, van de Velde CJH, Wouters M, Tollenaar R (2021) Preoperative risk factors for major postoperative complications after complex gastrointestinal cancer surgery: a systematic review. Eur J Surg Oncol 47(12):3049–3058 34340874 10.1016/j.ejso.2021.07.021

[25] Jeong O, Jung MR, Ryu SY (2018) Impact of various types of comorbidities on the outcomes of laparoscopic total gastrectomy in patients with gastric carcinoma. J Gastric Cancer 18(3):253–263 30276002 10.5230/jgc.2018.18.e27 PMC6160524

[26] He H, Ma Y, Zheng Z, Deng X, Zhu J, Wang Y (2022) Early versus delayed oral feeding after gastrectomy for gastric cancer: a systematic review and meta-analysis. Int J Nurs Stud 126:104120 34910976 10.1016/j.ijnurstu.2021.104120

[27] Tukanova KH, Chidambaram S, Guidozzi N, Hanna GB, McGregor AH, Markar SR (2022) Physiotherapy regimens in esophagectomy and gastrectomy: a systematic review and meta-analysis. Ann Surg Oncol 29(5):3148–3167 34961901 10.1245/s10434-021-11122-7 PMC8990957

[28] Gianotti L, Fumagalli Romario U, De Pascale S, Weindelmayer J, Mengardo V, Sandini M, Cossu A, Parise P, Rosati R, Bencini L, Coratti A, Colombo G, Galli F, Rausei S, Casella F, Sansonetti A, Maggioni D, Costanzi A, Bernasconi DP, De Manzoni G (2019) Association between compliance to an enhanced recovery protocol and outcome after elective surgery for gastric cancer. Results from a western population-based prospective multicenter study. World J Surg 43(10):2490–2498 31240434 10.1007/s00268-019-05068-x

